# A case of mediastinal hyperparathyromatosis

**DOI:** 10.1093/jscr/rjad735

**Published:** 2024-01-18

**Authors:** Chloe Spillane, Gavin Calpin, Sneha Singh, Kasie O’Reilly, Cian Hehir, Arnold Hill, Colm Magee, Helen Barrett

**Affiliations:** Department of Surgery, Beaumont Hospital, Beaumont Road, Beaumont, Dublin 9, D09V2N0, Ireland; Department of Surgery, Beaumont Hospital, Beaumont Road, Beaumont, Dublin 9, D09V2N0, Ireland; Department of Surgery, Beaumont Hospital, Beaumont Road, Beaumont, Dublin 9, D09V2N0, Ireland; Department of Surgery, Beaumont Hospital, Beaumont Road, Beaumont, Dublin 9, D09V2N0, Ireland; Department of Surgery, Beaumont Hospital, Beaumont Road, Beaumont, Dublin 9, D09V2N0, Ireland; Department of Surgery, Beaumont Hospital, Beaumont Road, Beaumont, Dublin 9, D09V2N0, Ireland; Department of Medicine, Beaumont Hospital, Beaumont Road, Beaumont, Dublin 9, D09V2N0, Ireland; Department of Pathology, Beaumont Hospital, Beaumont Road, Beaumont, Dublin 9, D09V2N0, Ireland

**Keywords:** recurrent hyperparathyroidism, ectopic parathyroid, mediastinal parathyroid nodule, parathyromatosis, hyperparathyromatosis

## Abstract

Recurrent hyperparathyroidism (HPT) after initial parathyroid surgery occurs rarely in an ectopic location. The rare phenomenon of parathyromatosis may be the cause of this. We present the case of a 59-year-old woman with recurrent HPT, which presented as a new ectopic mediastinal parathyroid gland 13 years after initial 3.5 gland parathyroidectomy. A 1.5 × 1.3 cm lesion was discovered as an incidental finding in the pretracheal region, closely abutting the aortic arch. An aspirate revealed oncocytic cells, which were positive for parathyroid hormone, confirming a mediastinal parathyroid nodule. Sestamibi scan confirmed an avid nodule in the mediastinum. This patient had multiple co-morbidities but was asymptomatic of HPT. It was therefore decided at multi-disciplinary team discussion that she should undergo surveillance. To our knowledge, no such presentations have been reported in the literature. Thus, our case report is a unique addition of an atypical presentation of HPT.

## Introduction

Hyperparathyroidism (HPT) is characterized as abnormal calcium and phosphate metabolism caused by excessive secretion of parathyroid hormone (PTH). In tertiary HPT, an excess of parathyroid hormone is secreted by parathyroid glands, usually after longstanding secondary HPT. Some authorities reserve the term for secondary HPT that persists after successful renal transplantation [1, 2]. Conservative medical management including calcimimetics are usually attempted first before surgery is considered [3].

Ectopic parathyroid glands involve the atypical location of a parathyroid gland resulting from aberrant migration during early development. The prevalence of ectopic parathyroid glands varies greatly in recent literature, ranging from 6.3% to 52% based on cadaveric studies [4]. The mediastinal site is the rarest and accounts for 10%–15% of ectopic parathyroid adenomas [5, 6]. In addition, parathyromatosis is a rare but challenging aetiology of recurrent HPT. Parathyromatosis is the presentation of several nodules of benign hyperfunctioning parathyroid tissue scattered throughout the mediastinum and neck [7].

## Case report

We present a 59-year-old woman with complex HPT 13 years after a 3.5 parathyroid gland exploration. She had a background of colorectal cancer with a subtotal colectomy and ileo-sigmoid anastomosis. She subsequently underwent yearly surveillance computerized tomography of the thorax, abdomen, and pelvis (CT TAP). Two years after her diagnosis, a CT TAP revealed a new 1.5 × 1.3 cm lesion in the pretracheal region, closely abutting the aortic arch, not seen on previous imaging.

She had a complex medical history with autosomal dominant polycystic kidney disease, bilateral nephrectomy, end stage kidney disease for 12 years (dialysis for 4 years, then kidney transplant for 8 years then recently resumed dialysis), 3.5 gland parathyroidectomy 13 years previously, along with a recent myocardial infarction.

She proceeded with an endobronchial ultrasound (EBUS) and transbronchial needle aspirate (TBNA). However, this was non-diagnostic. A positron emission tomography (PET) scan showed a left thyroid mass, which was suspicious for malignancy along with adjacent FDG avid uptake. There was also a fluorodeoxyglucose (FDG) avid pretracheal node suspicious for metastasis. The repeat EBUS TBNA showed features of an oncocytic neoplasm. The initial immunoprofile was non-specific; immunostains for thyroid transcription factor 1 (TTF-1) and thyroglobulin were negative.

**Figure 1:**
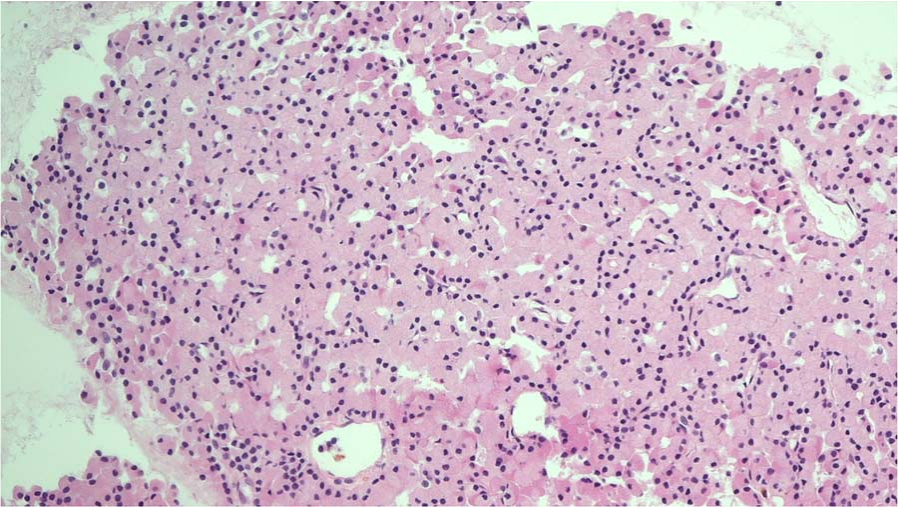
H&E stain of the FNA showing oncocytic cells with abundant eosinophilic granular cytoplasm from EBUS biopsy.

**Figure 2:**
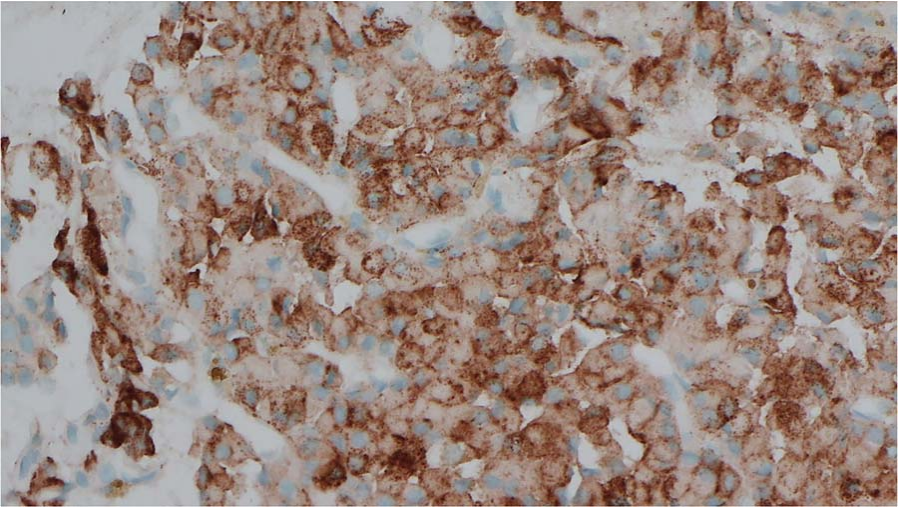
Immunocytochemical stain for PTH which is positive in the cells from EBUS biopsy.

**Figure 3:**
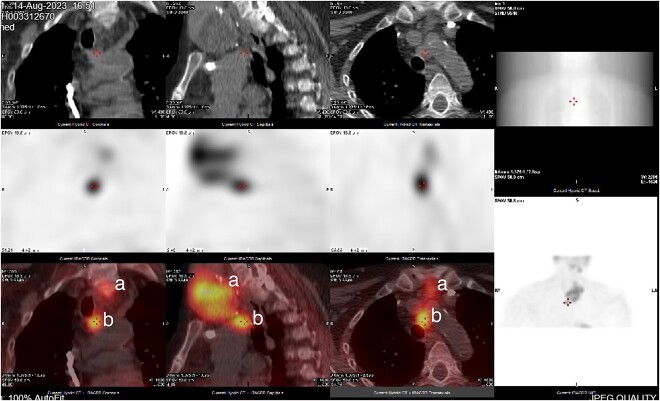
CT neck and sestamibi scan of coronal, sagittal, and axial view of superior mediastinum with mediastinal ectopic parathyroid nodule. (a) Thy3A thyroid nodule; (b) mediastinal parathyroid nodule.

She was subsequently referred to our service for further surgical evaluation. At the time of referral, she was asymptomatic. Initial blood tests showed an elevated PTH of 1256 pg/ml (range 15–65 pg/ml), reduced adjusted calcium of 2.05 mmol/l (range 2.21–2.52 mmol/l), normal free T4 at 19.9 μg/dl (range 12–22 μg/dl), and a reduced TSH <0.01 mU/l (range 0.27–4.20 mU/l), along with a raised phosphate of 2.32 mmol/l (range 0.81–1.45 mmol/l). A CT neck showed a large left thyroid mass with a differential diagnosis of metastatic lymphadenopathy versus a parathyroid adenoma. She underwent an ultrasound and fine needle aspiration of the thyroid mass, which revealed a Thy3A lesion with no evidence of malignancy. Following further multidisciplinary team (MDT) discussion, an immunostain for PTH was performed on the EBUS TBNA, which revealed that the oncocytic cells were positive for PTH (see Figs 1 and 2). This was confirmed by a sestamibi scan, which confirmed a sestamibi-avid nodule (see Fig. 3).

It was decided that the Thy 3A nodule did not warrant surgery as it was completely benign. In addition, there was a biopsy-proven mediastinal parathyroid nodule and given that this patient had significant co-morbidities including a recent myocardial infarction and was asymptomatic of her HPT, the consensus was that surveillance was considered the safest option. On follow-up, her PTH was 1100 IU, and calcium was 2.0 mmol/l. She was followed up in our clinic for surveillance thereafter.

## Discussion

In this case, we report an unusual mediastinal parathyroid adenoma 13 years after a 3.5 gland parathyroidectomy. Parathyromatosis is a rare cause of recurrence of HPT and accounts for only 21 cases described in the literature [8]. Parathyromatosis is defined as multiple nests of hyperfunctioning parathyroid tissue [9]. In 1977, Reddick *et al*. proposed two theories to explain the pathogenesis [9]. First, parathyromatosis is a consequence of spillage and seeding of parathyroid tissue within the operative field during parathyroid surgery. Second, preexisting parathyroid nests of embryological origin undergo hyperplasia under the influence of physiological stimuli. Parathyromatosis appears to be most prevalent in females with end-stage renal disease, which is consistent with our case [7]. The control and treatment of parathyromatosis remain a challenge with multiple attempts at surgical resection most often being unsuccessful [7].

Indications for parathyroidectomy in patients with tertiary HPT include severe hypercalcemia, persistent hypercalcemia, severe osteopenia, and symptomatic HPT [10]. Most abnormal mediastinal parathyroid glands can be removed by a cervical approach, which permits access to both the anterior and posterior mediastinum. Rarely, patients require a partial or complete median sternotomy [11]. Video-assisted thoracoscopic surgery is now widely used with an overall success rate of 98%–100% [12]. However, in this particular case, a neck which has had previous surgery poses significant technical challenges. Scar formation and distorted anatomy make it difficult to identify and safely remove abnormal parathyroid glands. It is critical that the operating endocrine surgeon has significant experience in both technique and judgement with risk and benefit being heavily weighted [13]. In addition, preoperative imaging plays a critical role in surgical planning as it obtains an adequate road map to guide the surgeon [14]. Interestingly, reported cure rate after reoperative parathyroidectomy varies from 87% to 93% [15].

This case emphasizes that care should be taken while handling parathyroid glands intraoperatively especially in the above co-morbid populations to avoid seeding and the cascade of scenarios to follow. In addition, this case also highlights the importance of preoperative imaging and balancing the importance of laboratory results, symptoms, and co-morbidities versus surgery.
